# Towards real-time communication between *in vivo* neurophysiological data sources and simulator-based brain biomimetic models

**DOI:** 10.1186/s40244-014-0012-3

**Published:** 2014-11

**Authors:** Giljae Lee, Andréa Matsunaga, Salvador Dura-Bernal, Wenjie Zhang, William W Lytton, Joseph T Francis, José AB Fortes

**Affiliations:** 1 Department of Electrical and Computer Engineering, University of Florida, P.O. Box 116200, 216 Larsen Hall, Gainesville 32611, FL, USA; 2 Department of Computer and Information Science and Engineering, University of Florida, P.O. Box 116120, E301 CSE Building, Gainesville 32611, FL, USA; 3 Department of Physiology and Pharmacology, State University of New York Downstate Medical Center, 450 Clarkson Avenue, Brooklyn 11203, NY, USA; 4 Department of Neurology, State University New York Downstate Medical Center, 450 Clarkson Avenue, Brooklyn 11203, NY, USA; 5 Department of Neurology, Kings County Hospital, 450 Clarkson Avenue, Brooklyn 11203, NY, USA; 6 Joint Program in Biomedical Engineering at Polytechnic Institute of New York University and State University of New York Downstate, 450 Clarkson Avenue, Brooklyn 11203, NY, USA; 7 Program in Neural and Behavioral Science at State University of New York Downstate, 450 Clarkson Avenue, Brooklyn, NY, 11203, USA; 8 The Robert F. Furchgott Center for Neural & Behavioral Science, State University of New York Downstate Medical Center, 450 Clarkson Avenue, Brooklyn, NY, 11203, USA

**Keywords:** Computational neuroscience, Neuroprosthetics, Brain-machine interfaces, Biomimetic models

## Abstract

Development of more sophisticated implantable brain-machine interface (BMI) will require both interpretation of the neurophysiological data being measured and subsequent determination of signals to be delivered back to the brain. Computational models are the heart of the machine of BMI and therefore an essential tool in both of these processes. One approach is to utilize brain biomimetic models (BMMs) to develop and instantiate these algorithms. These then must be connected as hybrid systems in order to interface the BMM with *in vivo* data acquisition devices and prosthetic devices. The combined system then provides a test bed for neuroprosthetic rehabilitative solutions and medical devices for the repair and enhancement of damaged brain. We propose here a computer network-based design for this purpose, detailing its internal modules and data flows. We describe a prototype implementation of the design, enabling interaction between the Plexon Multichannel Acquisition Processor (MAP) server, a commercial tool to collect signals from microelectrodes implanted in a live subject and a BMM, a NEURON-based model of sensorimotor cortex capable of controlling a virtual arm. The prototype implementation supports an online mode for real-time simulations, as well as an offline mode for data analysis and simulations without real-time constraints, and provides binning operations to discretize continuous input to the BMM and filtering operations for dealing with noise. Evaluation demonstrated that the implementation successfully delivered monkey spiking activity to the BMM through LAN environments, respecting real-time constraints.

## Background

Translation of our increasing knowledge of brain signals into treatment of patients with brain damage or with disconnections between brain and body requires the ability to read and transmit information bi-directionally between brain electrodes (or other probes) and a neuroprosthetic processor. This is the realm of brain-machine interface (BMI) and brain-computer interface (BCI), primarily distinguished by whether connecting to a relatively limited processor maintained on the body for managing a prosthetic or connecting to an external computer for the purpose of direct communication between the brain and a computer. It has been suggested that in the upcoming years, it will be important for neurosurgeons to understand and integrate BCI technology and its clinical applications into their field [1].

Currently, most of the algorithms utilized in BMI and BCI are based on low-dimensional interpretations of brain signals, typically based on rate determinations integrated over hundreds of milliseconds (ms) or seconds. By contrast, we are developing biomimetic brain models (BMMs) that attempt to replicate some of the attributes of brain signaling-specifically the spiking activation of the individual neurons. This increases the bandwidth requirements for communication since we now have the possibility of utilizing all the information from as many neurons as can be recorded (currently order 100) with firing rates of over 100 Hz. Learning can be incorporated into the BMM. This allows the hybrid BMM brain system to produce a coadapting symbiotic relation where both the brain and the BMM learn at the same time and adapt to each other [2].

Using data from a non-human primate, we are developing a test bed for the development of BMI/BCI neuroprosthetic rehabilitative solutions and medical devices for the repair and enhancement of neuronal systems. We have implemented BMMs capable of replicating many experimental paradigms, such as sensorimotor learning experiments [3–5] or cellular microstimulation [6,7]. They are able to accurately reproduce physiological properties observed *in vivo* [4,8], including firing rates and stimulus-induced modulations, and capture large-scale emerging properties such as local field potentials [9]. We have demonstrated that these BMMs, running in high-performance computers, can produce commands to control prosthetic devices in real time [10]. Here we aim at bridging the missing link by connecting the BMMs directly with animal electrophysiological recordings.

The proposed system is somewhat analogous to dynamic clamp [11], which is used to interface one or several single cells *in vitro* with a computer or analog device to simulate dynamic processes such as membrane or synaptic currents. However, scaling up the system to the next level, where a brain neuronal network is connected to biomimetic neuronal network model, posed a more challenging task.

Large-scale spiking neuron models of brain function are developed using neural network simulators such as NEURON, NEST, and GENESIS [12]. Existing BCI solutions do not support such simulator-based models. Instead, most existing solutions interface BCIs with artificial neural networks. Several general frameworks, systems, and software toolkits exist for this purpose: Virtual Integration Environment framework [13,14], BCILAB [15], BCI2000 [16], BioFeedback Software development Kit (BF++) [17], BCI++ [18], OpenVibe [19], BioSig [20] and Cyber-Workstation [21]. They usually support models developed in MATLAB, C++, or both of them. These tools help users to assemble and to conduct such computational modeling easily and efficiently for the BCI development providing reusable easy-to-use templates. However, in order to take advantage of these tools, models must be implemented or ported in specific languages supported by those tools and utilize simplified non-spiking neural network models.

In this paper, we address these issues by proposing a design and a prototype implementation for a network-based interface between an *in vivo* neurophysiological data source and a BMM that is simulating a network of spiking neurons. We specify the requirements of the system, detail the proposed design, and offer a prototype implementation following the design. The prototype links the PLEXON Multichannel Acquisition Processor (MAP) server, a commercial tool to collect signals from mi-croelectrodes implanted in a live subject, to a NEURON-based BMM of sensori-motor cortex that controls a virtual arm. Our implementation achieves low-latency interconnection between the data source and the spiking neuronal model.

## Methods

Since BMI systems also communicate via a processor, we will use the shorthand BCI to cover both BCI and BMI systems. In this section, we start by specifying the requirements of an interface system between neurophysiological data sources and BMMs. As discussed in the ‘Background’ section, this type of interface, which is not currently available, would be required to leverage the benefits of realistic large-scale spiking network models in BCI systems. We subsequently propose a generic or abstract design for a network-based system that meets the specified requirements. In the last subsection, we provide an example prototype implementation of the proposed generic design, which includes a more technical description of the different elements involved.

### System requirements

Generally, BCI systems must support three functions: data acquisition, data processing, and prosthetic control. In this way, system requirements of this study are similar to previous work in this area. In our case, the design must support the following generic functionality: (1) collection of empirical neurophysiological data from a live subject-person, monkey, or mouse-facilitated by data acquisition hardware; (2) delivery of collected data from the sources to the appropriate processing modules via network environments such as a local area network (LAN); (3) extraction of relevant information from the raw empirical data; (4) feeding of data into a BMM spiking neural network simulator, here NEURON [12]; and (5) support for both online and offline processing modes. These two processing modes are discussed further below as they are critical requirements of the system. This prototype does not deal with; (6) feeding of the BMM output into a physical prosthetic device (e.g., robot arm), which we have previously dealt with in a study of a real-time interface between a BMM and a robotic arm [10].

**Figure 1:**
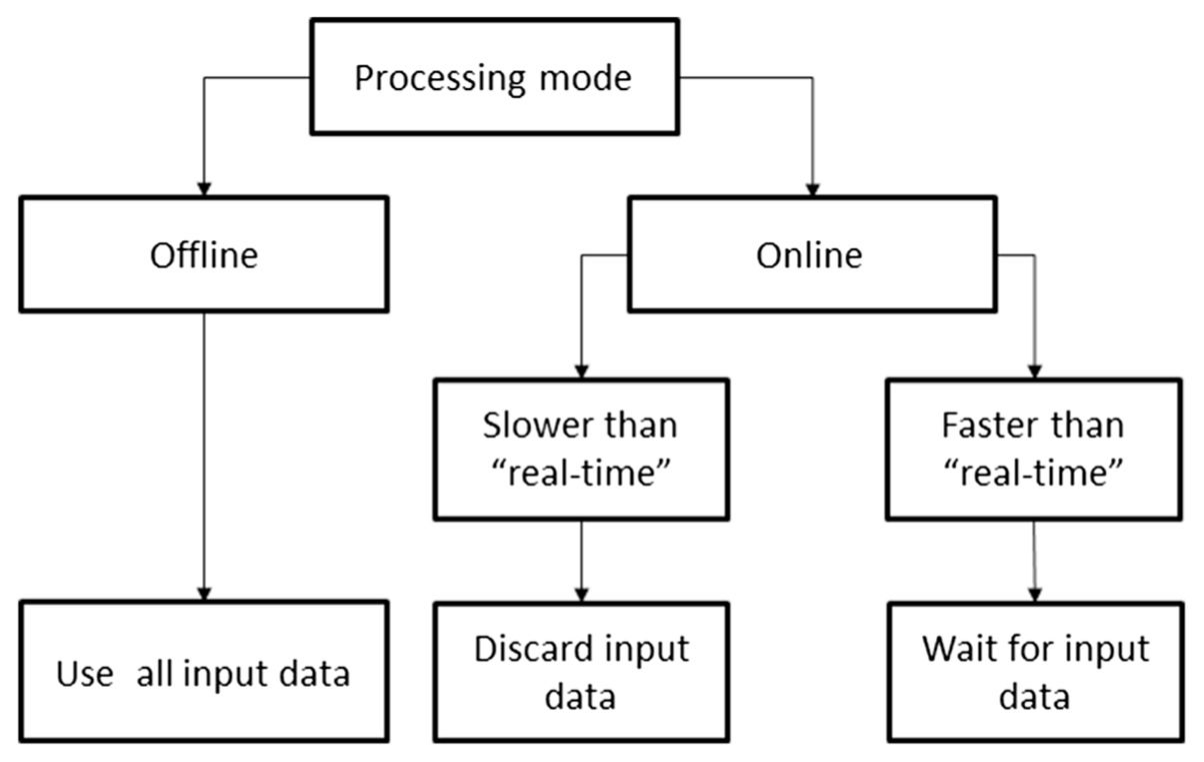
Requirements for the interaction between the data source and the BMM Requirements for the interaction between the data source and the BMM as a function of the simulation mode.

In the offline processing mode, all data delivered should be fed to the BMM without real-time constraints. Hence, all the data delivered should be kept in a queue so that the BMM can use the data as required. On the other hand, in the online processing mode, data delivery and simulation execution must meet real-time requirements. The BMM can run slower or faster than data acquisition, which occurs in real time. If the BMM runs faster than the generation of empirical data, it should wait for new input data to be received. If slower, the data delivered to the BMM must be partially discarded. Figure 1 illustrates how the offline and online modes should manage, under different circumstances, the interaction between the received neurophysio-logical data and the BMM.

### Design of a generic network-based interface

**Figure 2:**
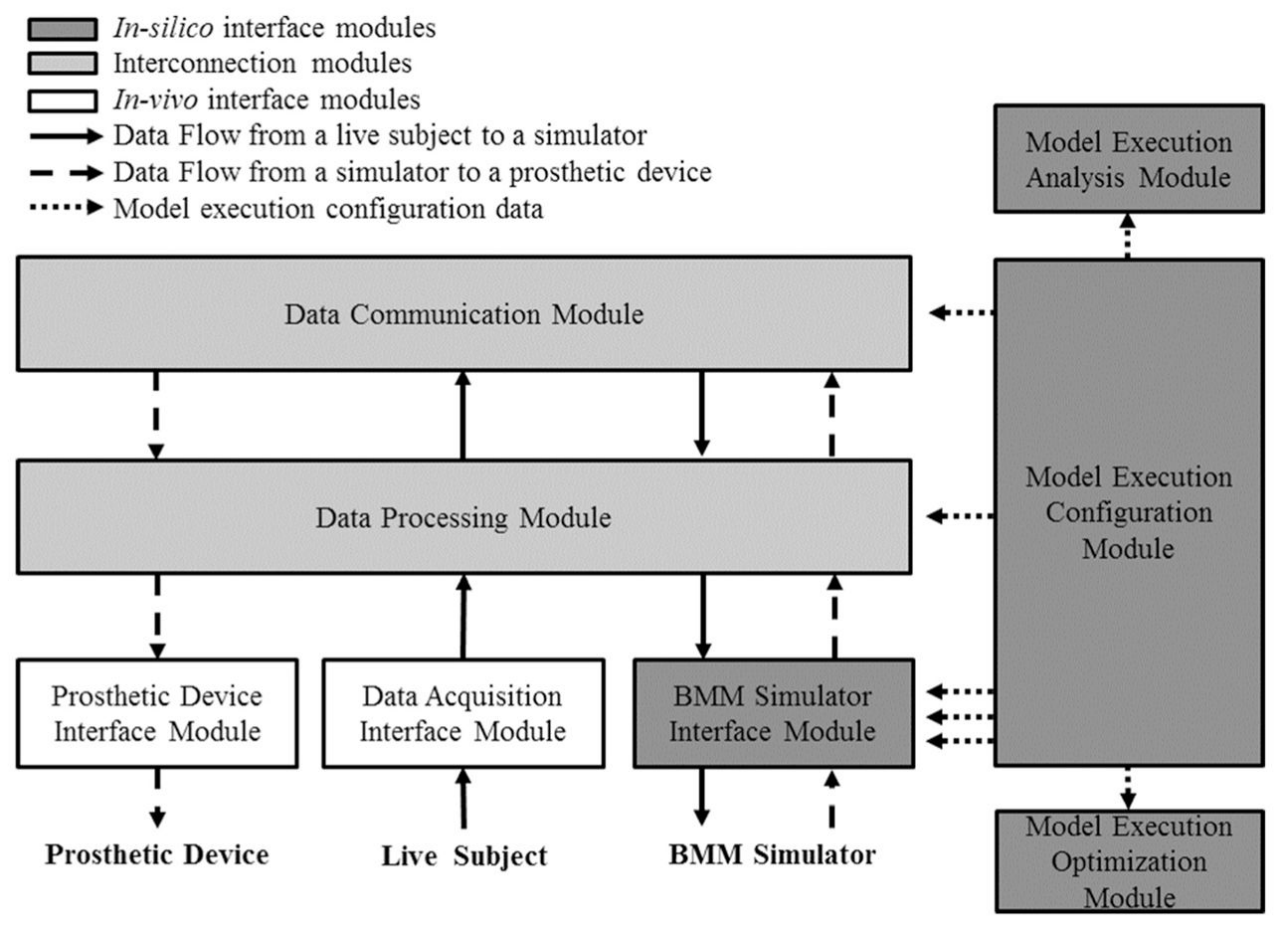
Modules structure and data flows of the design The design consists of three modules: *in silico* interface modules, interconnection module, and *in vivo* interface module. There are three data flows: from a live subject to a simulator, from the simulator to a prosthetic device, and from the model execution configuration module to other modules in order to set up the simulation environment with the user configuration parameters.

**Figure 3:**
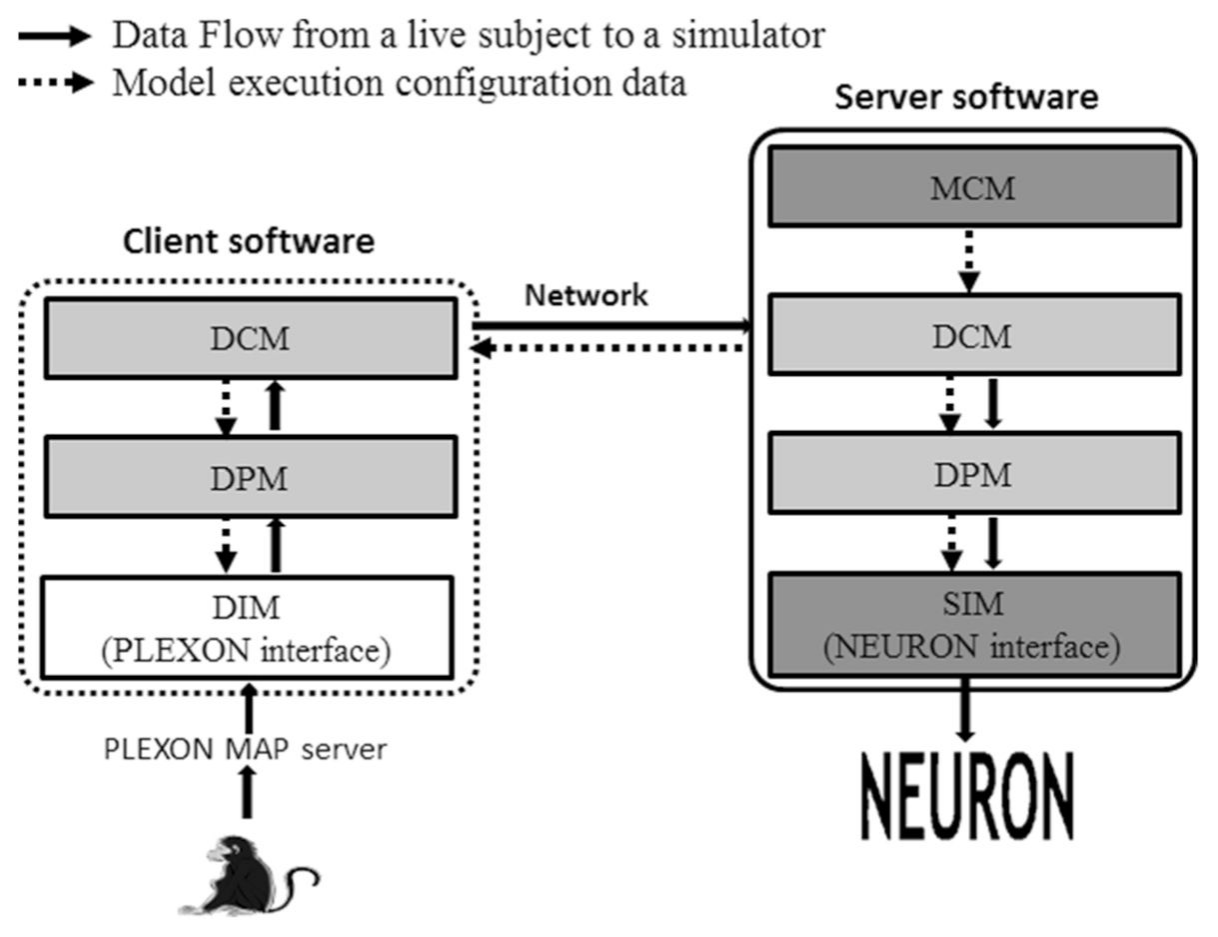
Logic diagram of the prototype implementation The arrows illustrate the flow for model configuration and for feeding data from a live subject to a BMM simulator. The targeted data source is the Plexon MAP server, and the BMM simulator is NEURON. Dotted arrows are the data flow for model execution configuration. Solid line arrows show the data flow from a live subject to NEURON.

We propose an abstract modular design capable of interfacing *in vivo* data sources with simulator-based neuronal network models, and more specifically, of satisfying the system requirements outlined in the previous section. Figure 2 illustrates the modular structure and data flow of the proposed design. Depending on the scenario, different instantiations of this abstract design are possible, one of which is provided as an example in the following section and depicted in Figure 3.

The generic design is composed of *in silico* interface modules, interconnection modules, and *in vivo* interface modules. The *in vivo* interface modules allow the system to interact with the data acquisition systems. The *in silico* interface modules allow the system to interact with the BMM, set the execution configuration parameters, and provide tools for model optimization and data analysis. The interconnection modules manage the information exchange between the *in vivo* and *in silico* interface modules. The design has three data flows. First, data collected from the subject via data acquisition hardware flows into the system through the *in vivo* interface modules. The interconnection modules then process and send this data to the *in silico* interface modules, where the data is fed as input to the BMM simulator. In the second data flow, simulation results are fed back into the system via the *in silico* interface and interconnection modules and are used to control a prosthetic device through the *in vivo* interface modules. The third data flow propagates the user configuration data to other modules in the design. The user configuration may include some important parameters for the interconnection of *in vivo* experiments and BMMs, such as the data transmission protocol and data processing options. Though the proposed design deals with interfacing to physical prosthetic devices [10], its integration into the framework is considered future work. Each module of the design is described in detail as follows:

#### Data acquisition interface module

There are many ways to record brain activity from a live subject. Here we focus on electrophysiology techniques such as single-unit recordings, which measure the changes in voltage of a single neuron; and local field potentials (LFPs), which provides a measure of the synaptic currents in a volume of tissue and have been previously used as input to a BMM [22]. Since different data acquisition methods and devices may generate data in different formats through different communication protocols, DIM is responsible for providing an interface between the neurophysiological data acquisition hardware and the rest of the system. The DIM delivers data received from a data acquisition device to the data processing module (DPM), which is discussed next.

#### Data processing module

The raw data (e.g., spikes) from the DIM may be fed directly into a BMM via the BMM simulator interface module (SIM) or may require additional operations to extract meaningful information, where the definition of ‘meaningful’ will vary depending on the destination of the BMM. The DPM is responsible for these additional operations. For example, the DPM may conduct filtering operations to the input data or perform operations to the results returned from the BMM simulation in order to convert them into commands that the prosthetic device understands.

#### Prosthetic device interface module (PIM)

This module delivers the commands converted from the simulation results as inputs to the prosthetic device. The type and format of the commands as well as the communication protocol required to transmit it will depend on the specific prosthetic device employed. Two examples of control command types for a robotic arm, a common type of prosthetic device, are *incremental* control (small differential changes to each joint of the robot arm) and *point-to-point* (specification of absolute endpoints).

#### BMM simulator interface module (SIM)

The SIM provides an interface to a BMM simulator to interact with the rest of the system. The SIM is responsible for providing bidirectional communication between the simulator and the data communication module (DCM), sending data processed by the DPM as input to the simulator, and delivering simulation results to PIM through the interconnection modules.

#### Data communication module (DCM)

In the design, it is critical to enable the transfer of data between the *in vivo* interface modules (DIM and PIM) and the module responsible for interfacing with the simulator (SIM) in real time. To minimize transfer latency, dedicated connections or LAN is recommended. The goal of DCM is to provide real-time data transmission between the *in vivo* and *in silico* interface modules. Prior to transmission by the DCM, data is processed by the DPM.

#### Model execution optimization module (MOM)

The MOM provides optimization functions that interact with the simulators during execution time, such as cell distribution methods for efficient balance and parallelism of large network models [23].

#### Model execution analysis module (MAM)

Some simulators have their own model analysis and visualization tools that users are already familiar with. However, the MAM allows the inclusion of additional ones, including simulation performance measurements such as CPU and memory usage. Users may access such results by calling functions provided by the MAM from their simulation code.

#### Model execution configuration module (MCM)

The MCM provides an easy-to-use configuration interface for setting parameters in all the modules in the design. For example, the user can select the most appropriate data communication protocol or data processing algorithm for a given experiment.

### Example prototype implementation

In this section, we introduce an example prototype implementation (Figure 3) of the proposed generic design (Figure 2). A client–server structure was used in our implementation. The path to feed data from a live subject to the BMM simulation is implemented using the following modules in the design: the MCM to allow the user to configure the system, the DIM to receive data from the Plexon MAP server, the DPM to process the collected data, the DCM to transmit the data via a LAN environment, and the SIM to feed the data to the NEURON simulator. PIM, MAM, and MOM are left as future work. The DIM (client) in the prototype is implemented in MATLAB to use MATLAB application programming interfaces (APIs) provided for interaction with the Plexon MAP server. The SIM (server) is implemented in Python so that it can interact with NEURON [24]. Figure 3 illustrates our implementation using a logic diagram, which is described in details below.

#### NEURON-based BMM

We tested the prototype implementation using a spiking neuronal network model of sensorimotor cortex [3,4]. The model can be trained to drive a simple kinematic two-joint virtual arm in a motor task requiring convergence on a single target by learning a sensorimotor mapping through reinforcement learning mechanisms. Individual neurons were modeled as rule-based dynamical units with many of the key features found in real neurons, including adaptation, bursting, depolarization blockade, and voltage-sensitive conductance [25,26]. The model consists of 288 excitatory and 64 inhibitory cells, each with AMPA, NMDA, and GABA synapses. These were arranged into three different populations with realistic and anatomical properties: 96 proprioceptive (P) neurons, representing specific joint angles; sensory neurons, which process inputs from the proprioceptive population; and motor neurons, which process input from the sensory cells and provide output to the virtual arm. The sensory population includes 96 excitatory sensory cells (ES), 22 fast-spiking sensory interneurons (IS), and 10 low-threshold spiking sensory interneurons (ILS). The motor population has 96 excitatory motor (EM), 22 fast-spiking motor interneurons (IM), and 10 low-threshold spiking motor interneurons (ILM). Cells are connected probabilistically with connection densities and initial synaptic weights varying depending on presynaptic and postsynaptic cell types. There is synaptic adaptation and training in the biomimetic model, but they do not require a training or validation dataset as they are based on the reinforcement learning paradigm. Synaptic weights are modified based on a global critic, which provides a reward or punishment signal depending on whether the army is moving towards or away from the target. After training, the model is evaluated by running the simulation and measuring the distance between the hand end point and target. More details about the BMM training and evaluation methods can be found in [4].

The model is able to provide two types of proprioceptive stimulus to the ES cells: through the P cells for continuous stimuli or through NetStim spike generator units with location (NSLOC) for discrete stimuli. P cells are implemented as integrate and fire neurons, and therefore include cell dynamics and internal variables, such as membrane voltage. When a spike is received from the external data source, an action potential is artificially triggered in the corresponding P cell. P cells also potentially allow for continuous stimuli inputs by modulating its membrane voltage over time. NSLOC units are simple spike generators, known as NetStim in the NEURON environment, with no internal dynamics. The user can determine its firing rate or, as in our case, the specific times when spikes should be generated. In our model, we have extended NetStim to have an x, y, z location (thus the name NSLOC) in order to facilitate distance-based random connectivity.

For the evaluation, two prerecorded monkey spiking data files from macaque primary motor cortex (M1) and dorsal premotor cortex (PMd) are fed to the BMM. The prototype implementation supports BMMs with continuous P-based and discrete NSLOC-based inputs. Thus, depending on the input cell type chosen, the discrete multiunit activity (MUA) values from the prerecorded spiking data are either processed by the binning operation as explained below for P cells or delivered directly as discrete input through the NSLOC units. P cell is a simple point process cell, so the binned input increases the voltage in the P cells, which makes the P cells fire once a threshold is reached, whereas NSLOC units generate spikes to corresponding ES cells at the times specified in the inputs.

#### Data acquisition interface module (DIM)

Plexon is a commercial tool for system neuroscience research. It collects signals from microelectrodes implanted in the brain of a live subject and provides MATLAB APIs for user software to connect with the Plexon system and to retrieve signals from it. The signals consist of channel number, unit number, signal type (e.g., event or spike), timestamp, and waveforms. The Plexon MAP server pulls signals from a data acquisition device and makes the signals available periodically (e.g., 10 ms). A user software can get the signals synchronously or asynchronously depending on APIs used (available at www.plexon.com). In the prototype implementation, DIM retrieves spikes from the Plexon MAP server by means of the synchronous API such that the Plexon MAP server notifies the DIM that spikes are available. The DIM then retrieves spikes as a chunk.

For the binning and filtering operations in the DPM described below, the fact that Plexon MAP server generates out-of-order spikes occasionally is taken into account, that is, the algorithm considers that the timestamps of all spikes in a chunk are not guaranteed to be greater than the timestamps of spikes in the previous chunks. Plexon offers its own offline analysis utilities to sort out-of-order spikes, but these do not work for online real-time processing. DIM executes a reordering operation in order to deal with out-of-order spikes and minimizes potential spike loss by adding a delay based on the maximum timestamp *K* present in the previous chunk. Larger values of *K* lead to less potential for spike loss but add delays that may impact real-time constraints. The choice of a data-driven value for *K* was based on empirical experiments that validated it as a good candidate to avoid loss in the offline mode and to avoid adding too much delay in the online mode. The reordering algorithm starts once the second spike chunk is received, and its output will be delayed, with respect to the input, by one spike chunk. Algorithm 1 describes the reordering operation.

**Algorithm 1:** The reordering operation

**Input:**
US, a set of unordered spikes.
RS, a set of spikes remaining in the previous reordering operation.
**Output:**
OS, a set of reordered spikes.
RS, a set of remaining spikes in this reordering operation.
**Process**:
TEMP←RSU US
RS’ ← spikes with timestamps greater than *K* in TEMP, where *K* is the maximum timestamp in US.
OS ←TEMP–RS’.

### Data Processing Module (DPM)

In our implementation, the DPM performs binning, itemizing and filtering operations. If the model requires continuous input, the binning operation is executed every single binning time window as defined by the user (e.g., 100 ms). The binning operation sums the number of spikes per microelectrode or channel for a given period of time, which leads to a spike frequency value per channel per bin. Algorithm 2 describes the binning operation:

**Algorithm 2:** The binning operation

**Input:**
IS, a set of spikes.
RS, a set of spikes remaining in the previous binning operation.
**Output:**
BS, a set of summed spikes per channel.
RS’, a set of remaining spikes in this binning operation.
**Process:**
TEMP←RSU IS.
BS ← {B_1_, B_2_, ..., B_n_}, where B_CH_ is number of spikes whose channel is CH, their timestamps are in the current binning time window in TEMP, and 1 ≤CH ≤ n.
RS’ ←TEMP-{spikes used for BS}

The PLEXON system acquires 96-channel MUA data recorded by the microelectrodes implanted in the monkey’s brain. Spikes from each channel are sorted according to their waveform shape into up to 4 units (1, 2, 3, and 4), with unsorted spikes in unit 0. Additionally, the system sometimes reported simultaneously occurring signals (sync spikes) on multiple electrodes within a very short interval (e.g., 25 microsecond (us)). Given that there are many noise sources, such as small displacements in the electrodes, we hypothesized that such unsorted or sync spikes can be regarded as noise that needs to be filtered out. Algorithm 3 describes the method for removing sync spikes.

**Algorithm 3:** Filtering sync spikes

**Input:**
IS, a set of spikes.
RS, a set of spikes remaining in the previous filtering operation.
T, sync time window (e.g., 25us).
**Output:**
FS, a set of spikes filtered.
RS’, a set of spikes remaining in this filtering operation.
**Process:**
TEMP ← RSU IS.
FS ← a set of spikes, where each spike exists solely in a sync time window, [T*p, T*(p+1)), p is an integer such that $\lfloor \frac{\mathrm{minimum}\;\mathrm{timestamp}\;in\;\mathrm{TEMP}}{T}\rfloor \leq p\leq \left\lfloor \frac{\mathrm{maximum}\;\mathrm{timestarmp}\;in\;\mathrm{TEMP}}{T}\right\rfloor$.
RS’ ← a set of spikes whose timestamps are in the sync time window, $\left[T*\left\lfloor \frac{\mathrm{maximum}\;\mathrm{timestamp}\;in\;\mathrm{TEMP}}{T}\right\rfloor ,\;T*(\left\lfloor \frac{\mathrm{maximum}\;\mathrm{timestamp}\;in\;\mathrm{TEMP}}{T}\right\rfloor +1)\right]$.

Since the binning and filtering operations require a certain amount of computation, it is important to be aware of where they are executed as this may disturb real-time processing. Depending on whether the operations take place in the DPM of the client or the server, our implementation provides two options: the Heavyweight Client mode (HWC) and the Lightweight Client mode (LWC). In the HWC mode, the operations are conducted in the client’s DPM while the server’s DPM just receives processed data from the client, and simply forwards them to the SIM. In the LWC mode, the client’s DPM just sends the raw spiking activity to the server’s DPM, where the binning and filtering operations are conducted.

For discrete input simulations, the server’s DPM performs the itemizing operation, instead of the binning operation, in both of the HWC and LWC modes, before pushing a chunk of spikes to the queue. The itemizing operation divides the chunk of spikes into groups, each with a fixed number of spikes (e.g., 20) for efficient queue management. For example, when the fixed number of spikes for a group is 20 and the DPM receives a chunk which has 30 spikes, the first 20 spikes in the chunk are pushed in the queue as a group. Then, another group composed of the remaining 10 spikes and another 10 spikes being assigned an arbitrary value (e.g., zero) is queued. A counter is used to track the number of real spikes so that the arbitrary valued spikes are never used. The time required for the itemizing operation is proportional to the size of chunk.

#### Data communication module (DCM)

The DCM supports two data transmission protocols: the User Datagram Protocol (UDP) and the Transmission Control Protocol (TCP). UDP is a connectionless transmission protocol that does not guarantee that packets sent reach the destination or that they are delivered in order [27]. On the other hand, TCP is a connection-oriented transmission protocol providing reliable and ordered transmission [28]. Ordinarily, TCP uses Nagle’s algorithm to buffer small packets in order to improve efficiency in transmission. Theoretically, the UDP transmission has less latency than TCP since UDP does not provide error checking for packets. On a LAN environment where packet loss occurs rarely, UDP is expected to deliver data without packet loss or reordering issues. However, UDP still can potentially lead to data being lost or out of order. The DCM provides both the UDP and TCP transmissions, and users can select the appropriate protocol according to the network environment. In the DCM, TCP Nagle’s algorithm was disabled since buffering small packets may disturb real-time communication. The client’s DCM was implemented in MATLAB using Java network communication APIs. Data formats are designed to operate with the data received from the *in vivo* recording system and may be reusable for different acquisition systems. Spike information from the Plexon MAP server is converted into the designed data format and delivered from the client to the server. The data format consists of a header and a payload field. The header field indicates the type of data packet (‘DATA’, ‘NODATA’, or ‘EXIT’). The payload field contains the binned data and the binning window number for the P-based model; and the timestamp, channel ID, and unit ID of each spike, for the NSLOC-based model.

#### BMM simulator interface module (SIM)

In order to feed the BMM running in NEURON with continuous or discrete input, inter-process communication between the SIM and the BMM was implemented with an existing first-in, first-out (FIFO) queue facility in Python. This queue provides easy-to-use operations such as putting/getting items into/from the queue and locking. For online mode simulations, the queue in SIM supports two cases: either the simulation is slower or faster than the rate of input of spiking data. The SIM assumes that input data is coming in real time. Therefore, in online mode, to recognize if the simulation is faster or slower than real-time, the SIM compares the timestamp in NEURON with the most recent input. If the difference is greater than a constant value, latency requirement (LR), which is fixed by user, the simulation is slow, so the SIM discards an item in the queue and executes the comparison again. Otherwise, the simulation is not slow and the item is processed. Discarding spikes allows the simulation to catch up with the data rate as less spikes are processed. We evaluate the consequences of this in the ‘Results’ section. For offline mode simulations, LR is set to a value bigger than simulation time (e.g., 1,000 s).

To deliver input from the queue to the BMM, we utilized a callback function that is added to the NEURON event queue and invoked at a specific simulation time by NEURON. When the function is invoked, it fetches data in the queue and then feeds the data as input to the BMM. Since the SIM queue contains only valid input data (ordered, filtered, and complying with real-time constraints), the callback function just pulls an input from the queue (if one exists), feeds it to the BMM, and moves the simulation forward. When the queue is empty, the function waits for data to arrive.

#### Model execution configuration module (MCM)

The MCM provides an initial handshaking protocol to establish connections between the client and the server and between the client and Plexon MAP server. Through the initial handshaking protocol, the client and server share configuration information such as the transmission protocol and where the binning operation takes place. Therefore, users only need to change parameters or flags in the configuration file located on the server side. Further details on the parameters found in the configuration file and how they can be modified by the user are described in [29].

#### Computational environment

For demonstration of the prototype implementation, the server and client ran in different machines connected to each other via a LAN environment. We constructed two system environments: ENV1 and ENV2. ENV1 is composed of two identical machines to conduct fair comparison of performance between the HWC and LWC modes. ENV2 consists of machines with better performance than those in ENV1 and arranged in a realistic research environment, with the machines located in two distinct research laboratories, the *in vivo* and *in silico* labs, and connected via LAN. The Plexon server emulated the *in vivo* data acquisition on the same machine where the client (DIM) ran. It retrieved spiking activity from prerecorded Plexon files. Table 1 describes the two computational environments in detail.

Round-trip time (RTT) was measured between two physical machines running the client and server in ENV1 and ENV2 varying the size of data transmitted (ranging from 64 to 1,032 bytes) according to the processing modes and datasets used in later experiments. The RTT was measured with the Linux Ping command using S option for specific data sizes. The RTT serves as a baseline to analyze whether or not the prototype implementation achieved expected transmission time. As the size of transmitted data increased (from 64 to 1,032 bytes), RTT also increased in both ENV1 (from 0.71 to 0.87 us) and ENV2 (from 0.98 to 1.55 us). RTT in ENV2 was greater than in ENV1 for all data sizes, with the largest difference observed for a data size of 1,032 bytes (1.55 vs 0.87 us).

For the experiments, we used two datasets recorded by the Plexon system, which were extracted from a live macaque monkey’s M1 and PMd regions for 100 s of real time. The macaque monkeys were performing a center-out reaching task (distance to targets was 4 cm, target radius was 0.8 to 1 cm), with her right arm attached to exo-skeletal robotic manipulandum (KINARM), corresponding to the initial manual training described in [30]. The animal had been implanted (after reaching task proficiency level of approximately 80% success) in M1 and PMd, representing the right shoulder and elbow regions, with multiple ‘Utah’ microelectrode arrays (10 × 10 electrode grid). The total number of units in the data was 185 and 97 for M1 and PMd, respectively. However, at this point, the specifics of the spiking data are not determined since the model is not yet employing it to learn a task; instead, the focus of the paper is on putting forward a proof-of-concept of the real-time interface. The datasets were selected to be representative examples of physiological inputs that can be potentially exploited by the biomimetic model.

## Results

We conducted a series of experiments to verify that the prototype implementation succeeded in enabling the interaction between the *in vivo* data source and the simulator-based sensorimotor cortex BMM under the principles of the proposed design. Using the two datasets on the two different computational environments, we measured the overhead caused by modules between the NEURON simulator and the Plexon SoftSer-ver. In these experiments, the overheads included the latencies for the following operations: (a) reordering, (b) binning (for continuous input) or itemizing (for discrete input), (c) filtering, (d) transmitting binned or chunk of spikes, (e) pushing binned (or chunk of) spikes to the queue, and (f) pulling from the queue to feed the BMM.

Assuming the binning operation requires *n* chunks of spiking data, the latency in the binning operation is calculated as the time interval between the reception of the *n*th chunk of data by the DPM and the completion of binning computation. The transmission latency is the time required to deliver a chunk of spiking data or a binned spikes set through the network. For the latency measurement, we used a simplified version of the BMM; while accepting continuous input through P cells or discrete input through NSLOC units, P cells and NSLOC units were disconnected from the ES cells to prevent model computations from taking place. For both the offline and online processing mode simulations, we ran the full version of the NEURON model for 100 s of simulated time. We also measured the elapsed time, which corresponds to the execution time of the run phase in NEURON. Note that experimental results on ENV1 were about the same as the ones on ENV2 regardless of which transmission protocol between UDP and TCP was used. Hence, for the latency measurements, only the TCP results on ENV2 are presented in this paper. In terms of execution time and spikes generation, we only report the results from the HWC mode simulations, given that the LWC mode simulations results were almost identical, both in ENV1 and ENV2.

Table 2 shows the number of spikes after applying various filtering options provided in the prototype implementation and the percentage comparison with the no filtering case (no filter). The following shows the filtering options in the implementation: no filter; filter of unsorted spikes (unsort); filter of sync spikes within 25 us (25 us window); filter of unsorted spikes that are in 25 us sync window (unsort 25 us); filter of sync spikes within 1 ms; filter of spikes unsorted as well as in 1 ms sync window (unsort 1 ms).

**Figure 4:**
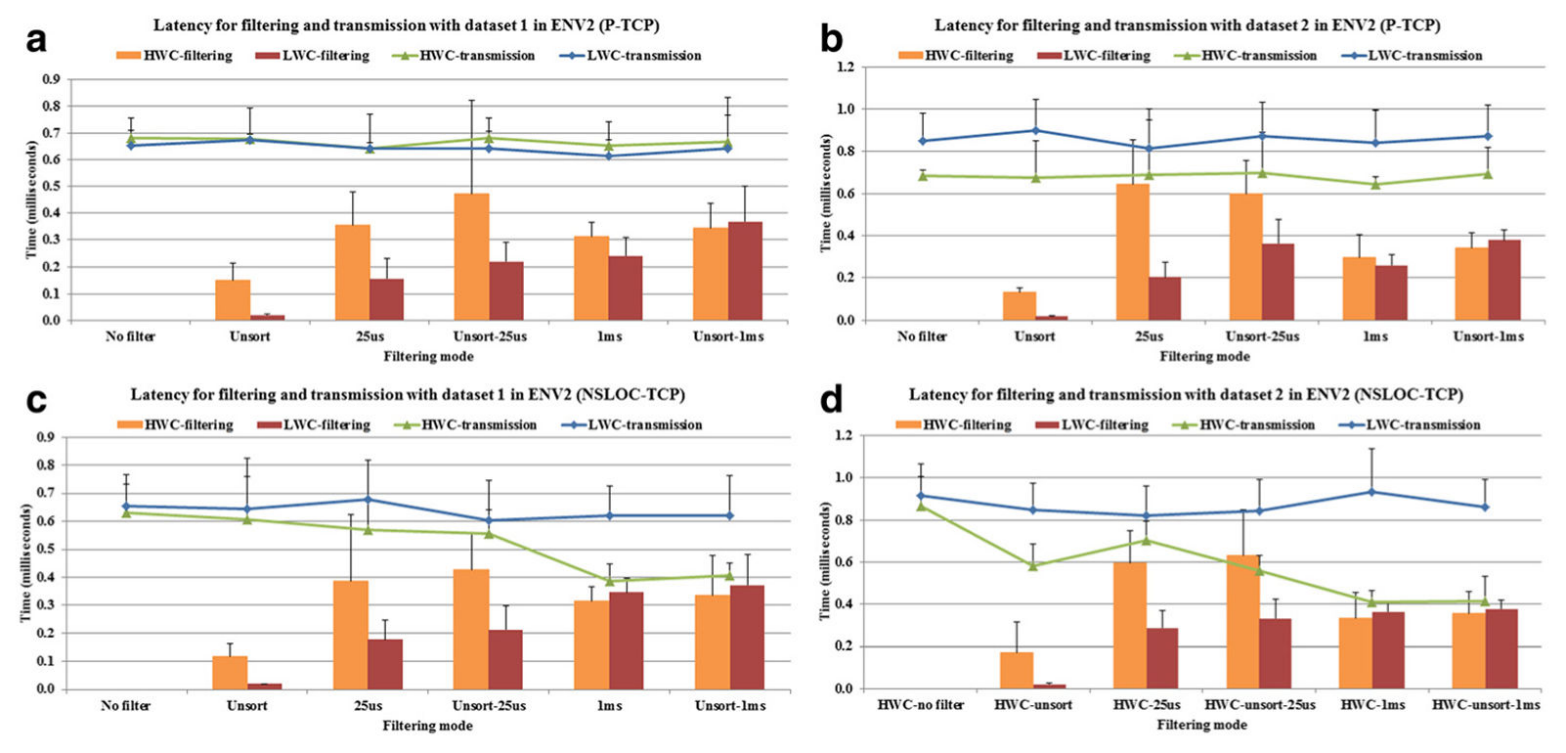
Latency for filtering and transmission in ENV2 with TCP transmission as a function of filtering options **(a)** The P-based model with dataset 1; **(b)** The P-based model with dataset 2; **(c)** The NSLOC-based model with dataset 1; **(d)** The NSLOC-based model with dataset 2.

Figure 4 shows the filtering and transmission latency in ENV2 for different filtering options. For the P-based simulation with the HWC mode, the packet size (payload) of the binned data is always fixed to 776 bytes (96 channels × 8 bytes + 8 bytes per binning window). On the other hand, with the LWC mode, the size of the chunks sent by the client varies with an average of 600 bytes for dataset 1 and 1 Kbyte for dataset 2. This difference of packet size between the HWC and LWC mode causes the HWC mode transmission time to be either similar (Figure 4a) or lower (Figure 4b) than that of the LWC mode in the P-based simulations.

Due to the difficulty of synchronizing the time in two distinct machines, we measured overhead by asking the client and the server to send very small-sized messages before and after each operation to a time collection server running in another machine in the same LAN environment. Therefore, considering the overhead caused by the measurement process, the prototype implementation achieved reasonable transmission times (<1 ms). As shown in Figure 4c,d, for the NSLOC-based simulation, where the client just sends chunk of spikes to the server, the HWC mode shows lower latencies than the LWC mode, specially for the 1 ms filtering options, as it benefits from having to transmit less data.

Figure 4 also shows that the filtering operation in the LWC is faster than in the HWC mode except for 1 ms and unsort 1 ms options. This suggests that the filtering operation in the LWC mode, implemented in Python, performs better than in the HWC mode implemented in MATLAB, when filtering is based on individual spikes comparison (25 us). However, there is a little difference between implementations for filtering based on group based comparison (1 ms).

Table 3 shows the total latency in ENV2 as a function of the different filtering options supported by the prototype implementation. Total latency includes the time for the reordering, binning for P (or itemizing for NSLOC), filtering, transmission, pushing, and pulling operations. For dataset 1, the total latency in the LWC mode simulations is generally lower than in the HWC mode, regardless of filtering options. However, in dataset 2, this difference is not so clear, which overall means that it is not possible to conclude which mode is better: factors such as the spike frequency and spike pattern have a significant influence on the total latency.

The size of a single queue item for the NSLOC-based simulation is 496 bytes, composed of 20 spikes × 3 elements per spike × 8 bytes, 8 bytes for the number of spikes in the item, and 8 bytes for the serial number of the item. For the P-based simulation, the size is 776 bytes (96 channel × 8 bytes + 8 bytes for binning time). However, the pushing and pulling operations in the NSLOC-based simulations take significantly more time than in the P-based ones. In the prototype implementation, there are two ways for the queue to recognize whether the simulation is faster or slower than the real time. First, the queue checks how many items it contains. If it has less than two items, it assumes that the simulation is running in real time. Otherwise, the queue carries out an additional test to check the simulation: it retrieves the current NEURON time through a shared variable, Python LOCK-based, and calculates the difference between the latest time in the queue items and the current NEURON time. If the difference is greater than LR, the simulation is considered to be running slower than the real time. Otherwise, the queue assumes that the simulation is running in real time. For the latency measurement, we used a simplified model; hence, the queue in the P-based model is likely to have a single item within a binning interval, which prevents the queue from fetching the current NEURON time. However, in the NSLOC-based model, it is rare that the queue has less than two items, even for the simplified model, which increases its overhead for pulling/pushing from/to the queue.

**Figure 5:**
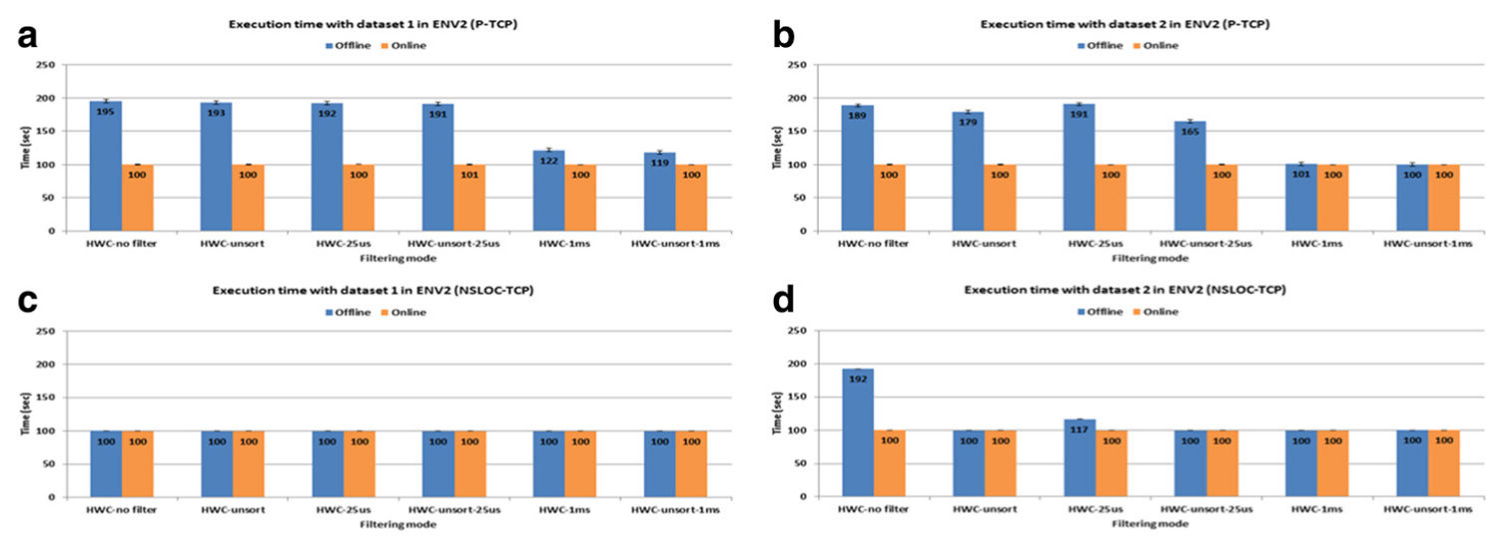
Execution time of the BMM in ENV2 with the TCP transmission Each simulation runs 100 s of simulated time. **(a)** The P-based model with dataset 1; **(b)** the P-based model with dataset 2; **(c)** the NSLOC-based model with dataset 1; and **(d)** the NSLOC-based model with dataset 2.

Figure 5 presents the execution time of the full version of the BMM in ENV2. The execution time in the P-based model with the offline mode is significantly above the 100 s required to meet the real-time constraint except when applying the HWC-1 ms and HWC-unsort-1 ms filtering options. On the other hand, most of the NSLOC-based simulations achieved real-time processing, even in the offline processing mode (Figure 5c,d). The offline processing mode with no filter option and dataset 2 takes about 1.9 times longer than with dataset 1. This is because the number of input spikes in dataset 2 without any filtering is about 2.3 times more than in dataset 1. Figure 5 also shows how the online processing mode in the prototype implementation satisfies the real-time constraints (approximately 100 s).

**Figure 6:**
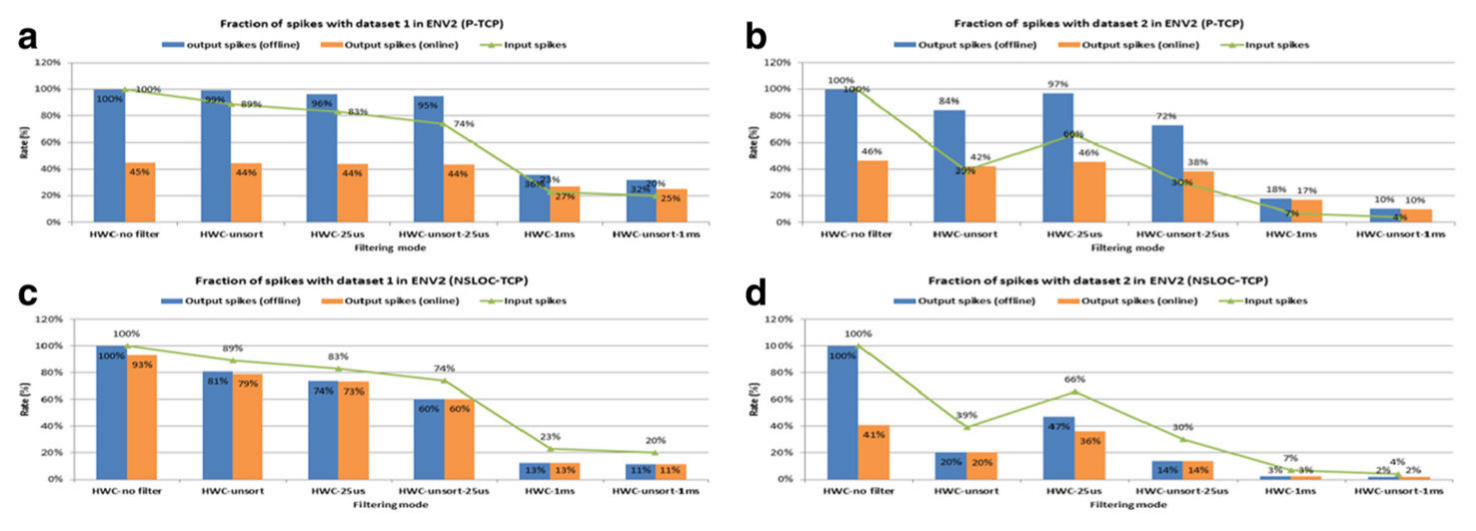
Fraction of input and output spikes for different processing modes The fraction of input and output spikes based on the offline processing mode simulation without filtering (HWC no filter) in ENV2 with the TCP transmission. **(a)** The P-based model with dataset 1; **(b)** the P-based model with dataset 2;**(c)** the NSLOC-based model with dataset 1; and **(d)** the NSLOC-based model with dataset 2.

Figure 6 presents the fraction of input and output spikes for each mode, taking as a reference the offline processing mode simulation without filtering (HWC no filter), which has 100% of both input and output spikes. As shown in the figure, all of the online processing mode simulations satisfy the real-time constraint. There are two factors that contribute to achieving the real-time constraint: filtering input sync spikes in the DPM and discarding input spikes in the SIM. When the filtering option is set to HWC no filter in the online processing mode, real-time processing is achieved only due to discarding input spikes in the SIM. If the number of output spikes of an online mode simulation is the same as that of the offline mode simulation, it means that only input spike filtering is playing a role in meeting the real-time constraints (e.g., HWC-unsort-1 ms in Figure 6b). Otherwise, the two methods are cooperating to deal with the real-time constraint.

In Figure 6c, the NSLOC-based online mode simulations with HWC no filter/unsort/ 25 us options generate slightly less output spikes than the offline mode ones with the same filtering options. This means that the online mode simulations discard some input spikes in the SIM. However, they should not need to discard any spikes given that the offline mode simulations with the same options are able to run in real time (Figure 5c). This happens because the prototype implementation was designed to keep the simulation moving forward even when there are no input spikes, so that the simulation achieves smoother arm trajectories. In order to do this, the callback function in the NSLOC-based online mode simulation checks the queue at very short intervals and forces NEURON to skip input spikes that are lagging behind real time. Therefore, the higher the input spike frequency is the more likely it is that some input spikes are behind the time the callback function is invoked. This explains why the three online mode simulations mentioned above, with a relatively high input spike frequency, have less output spikes than the equivalent corresponding offline mode simulations.

**Figure 7:**
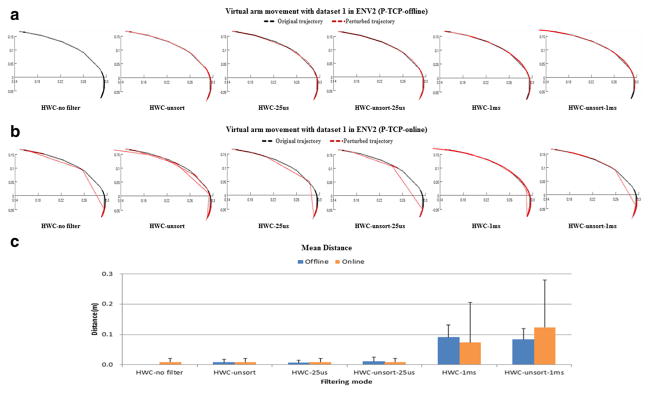
Virtual arm movement analysis as a function of filtering options for dataset 1 in ENV2 **(a)** Virtual arm trajectories in the P-based model with the offline mode; **(b)** virtual arm trajectories in the P-based model with the online mode; and **(c)** Euclidean distance between the trajectories generated by the different simulation modes and the reference trajectory, (averaged over 20 samples taken at 5 s intervals). The black dotted line shows the reference trajectories. The P-based model ran for 100 s of simulated time with the learning mode was turned off.

**Figure 8:**
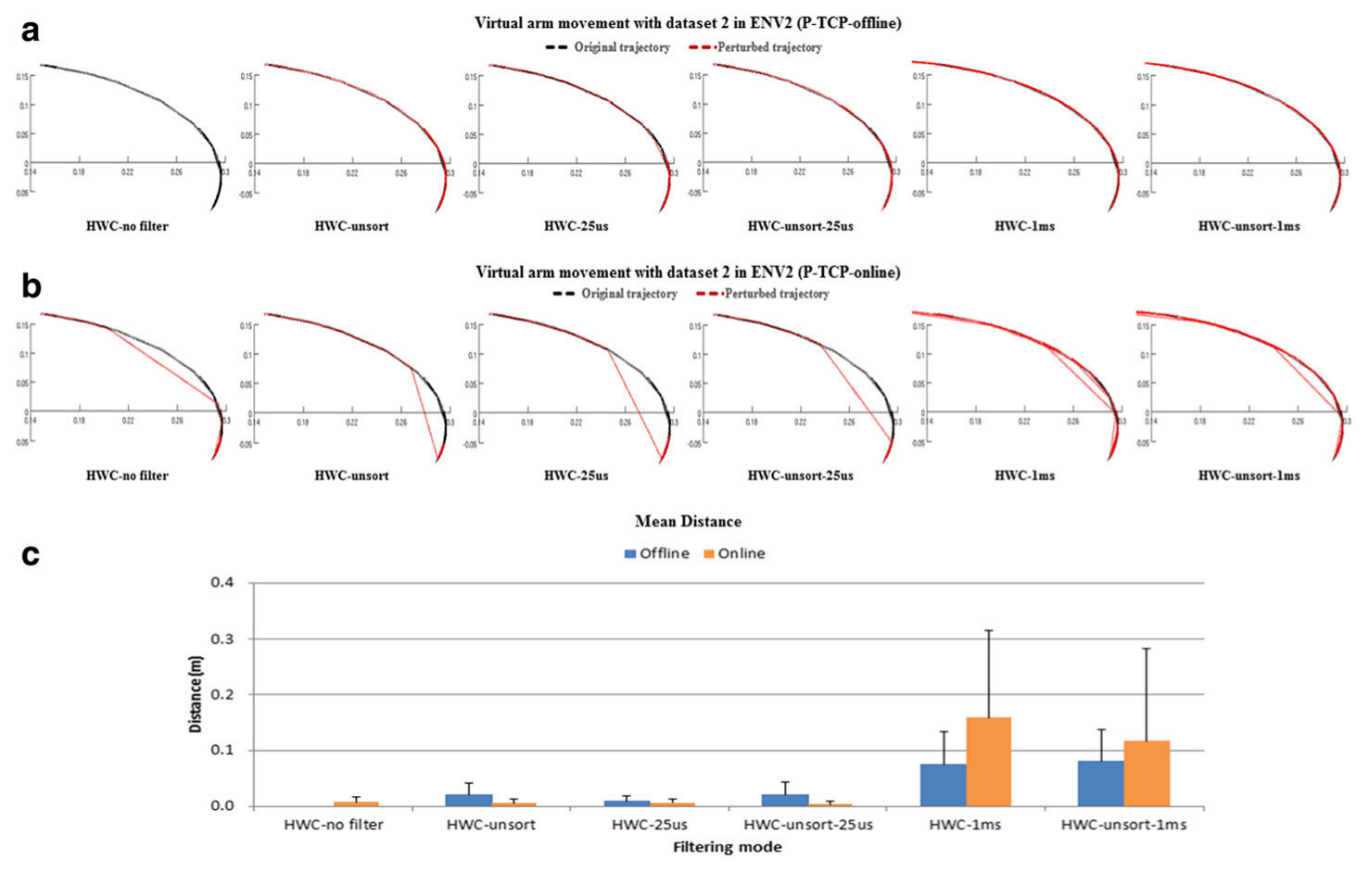
Virtual arm movement analysis as a function of filtering options for dataset 2 in ENV2 **(a)** Virtual arm movement in the P-based model with the offline mode; **(b)** virtual arm movement in the P-based model with the online mode; and **(c)** Euclidean distance between the trajectories generated by the different simulation modes and the reference trajectory (averaged over 20 samples taken at 5 s intervals). The black dotted line shows the reference trajectories. The P-based model ran for 100 s of simulated time with the learning mode was turned off.

Figure 7a,b represents the virtual arm trajectories, that is, the x-y location of the arm end-effector (hand) over time, generated by the P-based BMM simulations, for datasets 1 in offline and online modes, respectively. Figure 7c also shows the mean distance between each trajectory and the reference simulation trajectory (generated in the offline mode with no filtering and plotted in dotted black color), obtained by taking samples of the x-y hand position every 5 s. Figure 8 shows the results for dataset 2. The trajectories in the offline mode simulation are smoother than in the online mode because, as previously mentioned, in the online mode, the prototype implementation forces NEURON to skip its internal steps when the simulation is slower than real time. This leap causes the virtual arm to skip some movements, too. For example, the virtual arm trajectory graph in Figure 7a HWC no filter was plotted using 2,000 samples, whereas the trajectory in Figure 7b HWC no filter consisted only of 498 samples. Figures 7 and 8 show that, except for the 1 ms and unsort 1 ms filtering options, both the offline and the online mode simulations are accurately generating virtual arm trajectories very similar to that of the reference simulation.

## Discussion

The prototype implementation achieved transmission between the data source and the BMM with low overhead (<3.5 ms). This was achieved through the use of basic Java-based MATLAB and Python-based libraries. Python threading is limited by its global interpreter lock [31]. Hence, we conducted the real-time processing by using Python processes and inter-process communication with NEURON. Commonly used neuronal simulators such as NEURON [24], NEST [32], GENESIS [33], and MOOSE [34], for which BMM spiking network models have been implemented, all provide Python-based interfaces. Being Python-based, the interface with NEURON can be retargeted to other simulators that use a Python interface and offer callback functions. In addition, the binning and filtering operations were implemented for both the HWC and LWC mode, i.e., in MATLAB and Python. As demonstrated, the HWC and LWC modes have similar latency in the two given environments. In other scenarios, users can select the mode that suits better their system performance as needed.

The prototype implementation of the design currently enables the data flow from the Plexon MAP server (DIM) to NEURON (SIM). Depending on the data acquisition devices and BMM the spiking network simulators used, it might be necessary to modify the implementation of modules to deal with different types of input and output, and different libraries. For example, if LFPs are to be used as input, then DPM and DCM could be extended to deliver this type of signal. Nonetheless, all operations were modularized to maximize module reusability in other contexts.

There are several inter-process communication methods available that support data streams, including sockets, message queueing, and pipes. The rationale for choosing the client–server model using TCP/IP socket communication for our prototype is its suitability for research in neuroprosthetics, where the different systems are typically interconnected in a LAN environment. The TCP/IP socket provides easy-to-use interfaces between different platforms (Windows and Linux) as well as different programming environments (MATLAB and Python), which are used in the prototype implementation. It is yet unclear what communication methods will be required for working neuroprosthesis of this kind in the future, but similar setups are a feasible option, for example, if the working neuroprosthesis is being used in a clinical environment [1].

With respect to TCP and UDP transmission comparison, it is generally known that the TCP transmission is slower than UDP [35]. Thus, previous BCI researchers utilized the ‘pure’ UDP, or used a modified UDP to increase reliability. In the prototype implementation, we applied the TCP transmission with disabled TCP Nagle’s algorithm and demonstrated that the TCP transmission was not inferior to the UDP transmission for the given data characteristics and LAN environments. Additionally, with the increase in spike processing time on the server, the LWC mode has a greater chance of missing spike chunks when using UDP. Hence, we suggest that this modified TCP transmission can be used instead of UDP and still achieve reliable spike transmission in LAN environments.

In the prototype implementation, the online mode simulations generated significantly less spikes than the offline mode ones since a large number of input spikes were discarded by the queue in the SIM. The queue is aimed at achieving real-time simulations while minimizing the discarding of input spikes but does not consider the type of spikes being discarded or the effect this has on the network. In addition to the queue, we devised an input spike filtering operation, based on the assumption that unsorted spikes or spikes firing simultaneously within a short sync time window (e.g., 25 us) were the result of noise in the system. As shown in Figures 7 and 8, even after removing these sync spikes, the BMM-driven virtual arm tracked the reference arm trajectory. This worked in both offline and online modes, except for two of the filtering cases: HWC-1 ms and HWC-unsort-1 ms. Because experiments have not yet revealed the nature of the simultaneously occurring spikes, we cannot definitively conclude they were caused by noise. However, our results suggest that such input spikes did not play a critical role in the virtual arm movements.

Our design does not manage the computational resources required by the BMM simulators. This contrasts with previous methods, such as the Cyber-Workstation, which includes the parallel processing capabilities required for the concurrent execution of multiple models demanding large amounts of computing resources [21]. The design assumes that the BMM simulator will employ the necessary tools to manage its own computational resources. Nonetheless, achieving real-time processing may require parallelization of the BMM itself and efficient methods to feed *in vivo* data into a parallelized BMM.

In the present example, we assumed that the recorded MUA data represented pro-prioceptive information fed directly from the monkey’s brain to a corresponding neural population (P) for continuous input, or to spike generators (NSLOC) for discrete input to the BMM. These proprioceptive signals, which could potentially be recorded from M1, PMd or posterior parietal cortex (PPC), would represent the external state of the prosthetic device (e.g., joint angle) [36,37]. However, the focus of the paper is on demonstrating this external input can be provided in real time to the BMM; decoding the actual joint angles from the M1 or PMd data is out of the scope of this work. Recorded activity from M1 could also replace the background input noise currently used to drive the model, leading to more complex and realistic dynamics [22]. PMd activity has also been hypothesized to encode movement preparatory information such as the target to reach and path to follow [38]. Therefore, another potential application of our system is to use the target information encoded in the input PMd data to modulate the activity of the model towards reaching that specific target.

Recently, communication between a NEURON-based BMM and a physical robot arm was demonstrated in a LAN environment [10]. The outputs from the model were delivered to the physical robot arm via UDP transmission, with the robot arm moved following the received motor commands. However, in that study, the discrepancy in update frequency between the model, which run approximately 4 times slower than real time (approximately 6 Hz), and the robot arm (500 Hz) led to undesired abrupt trajectory changes. Making the model run in real time, by discarding input spikes or by filtering some of them, may be a possible solution to achieve a higher update frequency and therefore smoothen the robot trajectory. We are currently working on implementing additional data flow paths of the design, in particular, those to enable bidirectional communication between the NEURON-based model and a prosthetic device to form a closed-loop system.

## Conclusions

We proposed a design that enables communication between an *in vivo* data source, a simulator-based BMM, and a prosthetic device. The design specifies a set of modules and data flows among them. We implemented a prototype of the design, enabling interaction between the Plexon MAP server and a NEURON-based model of sensorimotor cortex capable of controlling a virtual arm. The prototype implementation showed that it supports real-time simulations with a low communication overhead. Interconnection between *in vivo* experiments and simulator-based BMMs opens the door to a new range of experimental paradigms in brain research. For example, the design could underlie hybrid experimental test beds combining brain activity and a BMM to develop new forms of brain-machine interfaces where the real and simulated brain coadapt and work together [2,36] in order to repair a damaged brain. Ultimately, the test beds could also be utilized to understand how neural-based cortical stimulation could be applied to accelerate recovery from brain injury [6,7,39].

## Figures and Tables

**Table 1:** Computational environments

Name	Resources	Client	Server
ENV1	CPU	Intel® Core^™^2 Duo CPU E840 3.00 GHz	Identical
	Memory (RAM)	4 GB	Identical
	OS	Windows 7 Enterprise	CentOS 6.4 (Final) running Linux kernel 2.6.32–358.el6.x86_64 #1 SMP
	Software	MATLAB R2013b (8.2.0.701) 64-bit (win64)	NEURON 7.4 (984)
		PLEXON SoftServer version 2.0	Python 2.7.3
ENV2	CPU	Intel® Core^™^2 Quad CPU Q8400 2.66 GHz	2 Intel® Xeon® CPU L5640 2.27 GHz (6 cores/CPU) with Hyper-threading enabled
	Memory (RAM)	6 GB	96 GB
	OS	Windows vista^™^ home premium 64-bit	Ubuntu running Linux kernel 2.6.38–8-generic #42-Ubuntu SMP
	Software	MATLAB R2007b (7.5.0.342) 64-bit (win64)	NEURON 7.4 (982+)
		PLEXON SoftServer version 2.0	Python 2.7.3

**Table 2:** Dataset analysis

Name	Dataset 1	Dataset 2
Data type	M1	PMd
Frequency (Hz) of input spikes	1,399	3,173

**Filtering option**	**Number and percentage of input spikes after filtering (% no filtering)**	

No filter	139,529 (100)	317,034 (100)
Unsort	124,011 (89)	122,725 (39)
25 us window (25 us)	116,314 (83)	207,525 (66)
Unsort 25 us	103,110 (74)	94,413 (30)
1 ms window (1 ms)	31,854 (23)	21,437 (7)
Unsort 1 ms	28,489 (20)	11,971 (4)
Total time (s)	100	100

**Table 3:** Total latency (ms) varying filtering options with the TCP transmission

Dataset		Dataset 1				Dataset 2			
Input type		P (continuous)		NSLOC (discrete)		P (continuous)		NSLOC (discrete)	
Client mode		HWC	LWC	HWC	LWC	HWC	LWC	HWC	LWC
Filtering option	No filter	1.79	1.59	2.35	2.23	1.75	1.86	2.81	2.73
	Unsort	1.91	1.59	2.35	2.29	1.84	1.98	2.25	2.54
	25 us window (25 us)	1.96	1.76	2.51	2.46	2.34	1.82	3.10	2.87
	Unsort 25 us	2.22	1.79	2.54	2.35	2.25	2.02	2.72	2.75
	1 ms window (1 ms)	1.90	1.57	2.10	2.21	1.77	1.86	1.87	2.44
	Unsort 1 ms	2.02	1.84	2.25	2.18	2.08	2.05	1.87	2.38

## Abbreviations

BCI: Brain computer interfaces
BMM: Brain biomimetic model
DCM: Data communication module
DIM: Data acquisition interface module
DPM: Data processing module
EM: Excitatory motor
ES: Excitatory sensory cell
FIFO: First-in first-out
HWC: Heavyweight client
ILM: Low-threshold spiking motor interneuron
ILS: Low-threshold spiking sensory interneuron
IM: Fast-spiking motor interneuron
IS: Fast-spiking sensory interneuron
LAN: Local area network
LFP: Local field potential
LR: Latency requirement
LWC: Lightweight client
M1: Macaque primary motor cortex
MAM: Model execution analysis module
MCM: Model execution configuration module
MOM: Model execution optimization module
MUA: Multi-unit activity
NSLOC: Modified NetStim for discrete stimuli
PIM: Prosthetic device interface module
PMd: Dorsal premotor cortex
PPC: Posterior parietal cortex
RTT: Round trip time
SIM: Simulator interface module
TCP: Transmission control protocol
UDP: User datagram protocol
